# Society for the Study of Inebriety

**Published:** 1893-07-15

**Authors:** 


### SOCIETY FOR THE STUDY OF INEBRIETY.

On Tuesday, the 4th inst., at a meeting of this Society, held at the Medical Society's Rooms in Chandos Street, a reception was given in honour of Sir Benjamin Ward

Richardson to congratulate him upon the honour recently conferred upon him by Her Majesty. The chair was taken bp Dr. Norman Kerr, President of the Society, who expressed the pleasure which was felt by all at this recognition of the great services to mankind rendered by Dr. Richardson. He only felt, with many others, that the honour might fitly have been bestowed 20 years ago. After alluding to Sir Benjamin's brilliant and useful career, and speaking of his successes in the literary, as well as in the scientific field, Dr. Kerr proposed a resolution of hearty congratulation, which was cordially responded to by Dr. George Hardy, Dr. Henty, Mr. Robert Rae, Mr. George Hilton, Dr. Bridgwater, Dr. Parsons, Dr. Danford Thomas, and others. In his reply, Sir B. W. Richardson said that Lord Byron spoke of himself as awaking one morning to find himself famous, and though he did not apply exactly that statement to his own case, he ventured to say that on the day when this honour was graciously conferred upon him by her Majesty he awoke to find himself beloved by his countrymen and women beyond any conception he could possibly have formed of their good-will or esteem. Congratulations from friends new and old, rich and poor, had come to him. Briefly reviewing his medical career, Sir Benjamin concluded by saying that he craved no better reward for his labours than what sprang from the fervent hope that some day, however remote, his work, whether recognised or not, might be of some use to humanity.

A beautiful basket of roses was presented to Sir Benjamin for Lady Richardson by Miss Norah Kerr. Afterwards a paper was read by Mr. J. R. McIlraith on "Proposed Changes in the Law relating to Habitual Drunkards."

